# Can we predict severity of intrahepatic cholestasis of pregnancy using inflammatory markers?

**DOI:** 10.4274/tjod.67674

**Published:** 2017-09-30

**Authors:** Çiğdem Yayla Abide, Fisun Vural, Çetin Kılıççı, Evrim Bostancı Ergen, İlter Yenidede, Ahmet Eser, Oya Pekin

**Affiliations:** 1 University of Health Sciences, Zeynep Kamil Women and Children’s Health Training and Research Hospital, Clinic of Obstetrics and Gynecology, İstanbul, Turkey; 2 University of Health Sciences, Haydarpaşa Numune Training and Research Hospital, Clinic of Obstetrics and Gynecology, İstanbul, Turkey

**Keywords:** Cholestasis, inflammation, mean platelet volume, platelet-to-lymphocyte ratio, neutrophil-to-lymphocyte ratio

## Abstract

**Objective:**

To investigate the association of inflammatory markers with severity of intrahepatic cholestasis of pregnancy (ICP).

**Materials and Methods:**

This retrospective case-control study was conducted with 229 pregnant women, 84 with ICP, and 145 age-matched healthy pregnant women. Patients were categorized as mild ICP (<40 µmol/L) and severe ICP (≥40 µmol/L) with regard to serum bile acids. Inflammatory markers (neutrophil-to-lymphocyte ratio (NLR), platelet-to- lymphocyte ratio (PLR) and mean platelet volume (MPV), and red blood cell distribution width (RDW) were compared between the groups.

**Results:**

Patients with ICP had significantly decreased RDW and increased white blood cell counts (WBC), MPV and PLR, but no significant changes in NLR. The comparison of mild and severe cases with regard to NLR, PLR, WBC, and RDW was similar (p>0.05). MPV levels were significantly increased in severe group (p<0.05).

**Conclusion:**

WBC, MPV, and PLR were the inflammatory markers significantly increased, and RDW was signifantly reduced in ICP. MPV was the marker that significantly increased with the severity of disease. The use of inflammatory markers in the assessment of perinatal outcomes needs further studies.

## PRECIS:

This paper investigated the hematological inflammatory markers in mild and severe cases of intrahepatic cholestasis of pregnancy. Mean platelet volume was significantly increased in severe intrahepatic cholestasis of pregnancy.

## INTRODUCTION

Intrahepatic cholestasis of pregnancy (ICP) is the most common liver disease seen during pregnancy, with a changing prevalence worldwide^(1,2)^. The etiology and pathogenesis of ICP are multifactorial. Environmental factors, nutritional deficiencies, hormonal changes, and genetic variations have been found to be responsible for ICP^(3,4)^. It presents most often in the form of pruritus in the second and third trimesters of pregnancy, with elevated serum aminotransferases and/or elevated serum bile acid levels (≥10 µmol/L)^(5)^. ICP can be differentiated from other types of liver diseases unique to pregnancy that share similar laboratory abnormalities such as preeclampsia, acute fatty liver of pregnancy, and hemolysis, elevated liver enzymes, and low platelet count (HELLP) syndrome^(1,6,7)^. In addition, other skin diseases that cause high transaminase levels in pregnancy must be excluded. After delivery, the symptoms of ICP usually resolve within 48 hours, with laboratory abnormalities normalizing within 2-8 weeks^(8,9)^.

Bile acid levels can affect perinatal outcomes and are related to an increased risk of iatrogenic preterm delivery, spontaneous preterm delivery, meconium-stained amniotic fluid (MSA), and sudden intrauterine death of the fetus^(8,10,11,12)^. In the severe ICP group, the incidence of complications is higher than in the mild group^(8)^. Unfortunately, ultrasonography, cardiotocography, fetal movements, and Doppler ultrasonography cannot predict fetal death; there is no perfect test or prognostic marker available to predict fetal outcome^(8,13,14)^.

Recent studies demonstrated the prognostic role of inflammatory markers in both cardiovascular diseases and malignancies^(15,16)^, but few studies have been performed with ICP. The neutrophil-to-lymphocyte (NLR) ratio, platelet-to-lymphocyte (PLR) ratio, mean platelet volume (MPV), and red blood cell distribution width (RDW) are hematologic inflammatory markers. One important pathogenesis responsible for the occurrence of ICP is inflammation; however, it is not known which mechanism initiates this inflammation^(17)^. Recently, NLR has been found to be a promising diagnostic marker in ICP^(18)^. To the best of our knowledge, no studies have investigated the roles of PLR and RDW in ICP. Therefore, we aimed to evaluate the role of inflammatory markers, which are readily available and easily calculated parameters, in the severity of ICP.

## MATERIALS AND METHODS

This retrospective case-control study was conducted at Zeynep Kamil Women and Children’s Health Training and Research Hospital, İstanbul. Patients with ICP who delivered their babies in this hospital from January 2013 to January 2016 were enrolled in this study. All data were obtained from hospital files and our computer database. This study was approved by the Zeynep Kamil Women and Children’s Health Training and Research Hospital Local Ethics Committee (approval number: 136).

The term ICP was used if the serum bile acid level was ≥10 µmol/L with pruritus that could not be explained by any other condition. A total of 102 women with ICP were enrolled in this study. The exclusion criteria were: patients with incomplete data, fetal congenital anomalies, multiple pregnancies, chronic/acute liver disease (Wilson’s disease, cholecystitis, primary sclerosing cholangitis, primary biliary cirrhosis, alpha-1-antitrypsin deficiency, symptomatic cholelithiasis, cytomegalovirus, Epstein-Barr virus, autoimmune hepatitis, or acute fatty liver of pregnancy), and HELLP syndrome. A total of 84 singleton pregnancies were included in this research.

The patients with ICP were categorized into two groups according to their serum bile acid levels: mild (<40 µmol/L, n=53) and severe (≥40 µmol/L, n=31). The control group was selected from age-matched healthy women who had singleton deliveries on the same day as that of patient’s with ICP. All gestational age-matched controls complied with the exclusion criteria (n=145).

Serum bile acids were evaluated using an enzymatic assay with intra and inter-assay precisions of 3% and 4%, respectively, [Diazyme Total Bile Acids (TBA) kit; Diazyme Diagnostic Laboratories, USA] and a Cobas C501 (Roche, USA). A blood analyzer (Cell-Dyn 3700; Abbott, USA) was used to determine the complete blood cell count (CBC). The CBC inflammatory markers measured were white blood cell counts (WBC), platelets, NLR, PLR, MPV, and RDW.

Perinatal death was defined as mortality from over 24 weeks’ gestation until 7 days postpartum. A low Apgar score was defined as a score of below 7 at 5 minutes. The main outcome of the measures was the association of the inflammatory factors (WBC, NLR, PLR, MPV, and PDW) with the severity of ICP.

### Statistical Analysis

The statistical analysis was performed using the Statistical Package for the Social Sciences for Windows version 18 (SPSS Inc., Chicago, IL, USA). A p value of less than 0.05 was accepted as being statistically significant, and all measurements were performed within a 95% confidence interval. The results of the study are expressed as means, standard deviations, and percentages. According to the data distribution, comparisons were made using Student’s t-test, ANOVA, or the chi-square (χ^2^) test, when appropriate. A post-hoc least significant difference test was used after the ANOVA analysis. Relationships between the data were evaluated using Pearson’s correlations.

## RESULTS

The comparison between the patients with ICP (n=84) and controls (n=145) showed similar ages (28±5.5 vs. 28.1±5.2 years, p>0.05) and gravidities (2.1±1.3 vs. 2.3±1.2, p>0.05). The ICP group showed significantly decreased gestational weeks at delivery (36.2±2.3 vs. 39.1±1.4 weeks, p<0.001) and birth weights (2899±623.3 g vs. 3373±413.9 g, p<0.001) when compared with the healthy controls. The comparison of the characteristic findings of the patients with mild and severe ICP is presented in Table 1. The majority of patients (77.3% mild ICP vs. 74.1% severe ICP) were overweight/obese and aged younger than 35 years (79.2% mild ICP vs. 70.9% severe ICP), respectively. The women with mild and severe ICP exhibited similar characteristics with regard to educational status, chronic disease history, and previous ICP history (p<0.05). Table 2 presents the laboratory findings; with the exception of serum bile acid levels, the women with mild and severe ICP had similar findings.

Serum bile acids were positively and significantly correlated with PLR (r=0.343, p=0.003), but the correlations of bile acids with WBC (r=-0.062), neutrophils (r=-0.198), lymphocytes (r=-0.112), MPV (r=0.08), RDW (r=-0.174), and NLR (r=-0.110) were statistically non-significant (p>0.05). Serum bile acids were negatively significantly correlated with gestational age at delivery (r=-0.390, p<0.001) and birth weight (r=-0.252, p=0.02), and PLR was negatively correlated with gestational age at delivery (r=-0.254, p=0.003).

The comparison of the obstetric outcomes in the women with mild and severe ICP is given in Table 3. The percentages of those having male fetuses, low Apgar scores, fetal distress, MSA, preeclampsia, perinatal/neonatal mortality, gestational diabetes, Rh isoimmunization, and abortus imminence were similar between the women with mild and severe ICP. However, the gestational age at delivery and time of diagnosis were earlier in the severe group (p<0.05). In addition, the cesarean section rate was significantly increased in the severe group (p<0.05).

Table 4 shows the comparison of the inflammatory markers between the normal and ICP groups. Overall, the inflammatory markers were significantly increased in the ICP group, including WBC, MPV, and PLR (p<0.05), and neutrophils, lymphocytes, and RDW were significantly decreased in the ICP group (p<0.05). However, NLRs were similar between the normal and ICP groups.

Table 5 presents the comparison of the inflammatory markers in the healthy controls and women with mild and severe ICP. The RDW (p=0.128) and WBC (p=0.535) values were similar between the women with mild and severe ICP. MPV was significantly increased in the severe ICP group when compared with controls and the mild ICP group (p<0.05). Despite the fact that PLR was increased and RDW was decreased in patients with ICP, they were not significantly changed between the mild and severe groups. MPV was the marker that significantly increased in the severe group.

## DISCUSSION

ICP is a liver disease of pregnancy that increases fetal mortality; therefore, early diagnosis and assessment of the severity of the disease is an important task. In this study, we aimed to investigate the associations between the readily available, but newly defined, inflammatory markers, NLR, PLR, MPV, and RDW, with the severity of ICP. The present study found that the inflammatory markers were significantly increased in patients with ICP, and that MPV increased with the severity of ICP.

Fetal distress, premature delivery, perinatal asphyxia, and intrauterine fetal death may all occur in patients with ICP^(2,8,19^), and increased bile acid levels are thought to be the cause of these complications^(20)^. Consistent with previous studies, we observed lower birth weights and more preterm deliveries among patients with ICP^(11,21,22,23)^. However, there were no significant differences found in maternal age, parity, diabetes history, history of chronic systemic disease, preeclampsia or maternal hepatitis B and C infections in the women with mild and severe ICP, which was consistent with the study by Kawakita et al.^(24)^. Some studies found similar cesarean section rates^(12,25,26)^, in contrast to others(20,27). In our study, cesarean rates were higher with severe ICP. Some authors found an increased MSA risk in the ICP group when compared with controls^(8,28,29)^, but some authors did not support these findings^(27)^. In addition, some previous studies reported an increased MSA risk in the severe ICP group when compared with the mild group^(26,27)^, but conflicting results have been reported^(29)^ in the literature. Also in our study, we didn’t find an increased MSA risk in the severe group when compared with the mild group. Fetal asphyxia in the newborns of patients with ICP has been reported frequently in the literature^(30,31)^. Overall, the characteristics of these patients show wide variations, and the findings are inconsistent in the literature^(30)^.

Previous studies about ICP and inflammation suggested that ICP was an inflammatory process, and that perinatal outcomes were related to inflammation^(18,31,32,33)^. Bile acids are thought to be related to inflammation, and they directly affect hepatocytes and stimulate the secretion of proinflammatory mediators, which causes neutrophil accumulation, extravasation, and activation^(17)^. Prior studies used the hematologic markers WBC^(18)^, MPV^(31,32)^, and NLR^(18)^ as inflammatory markers in ICP. However, as far as we know, no studies have investigated the relationships between ICP and PLR and RDW. Therefore, to the best of our knowledge, this paper is the first to determine the associations between ICP and all CBC inflammatory markers. The results showed that serum bile acids were positively and significantly correlated with PLR (r=0.343, p=0.003), and the ICP group had a significantly increased PLR and significantly decreased RDW ratio when compared with the controls. Despite the increased PLRs and decreased RDWs in the patients with ICP, they were not significantly changed in severe ICP.

Kirbas et al.^(18)^ found significantly higher mean WBC and NLR values and a lower lymphocyte count in their severe ICP group than in controls, and NLR was found to be even higher in patients with mild ICP. They also found a significant association between fasting TBA levels and NLR^(18)^. However, we found that neutrophil and lymphocyte counts were significantly decreased in patients with ICP; NLRs were similar between the normal and ICP group.

Platelets in the blood vary in size, with the granules and adhesion molecules of the platelets increasing when they become larger and play an active role in homeostasis^(34)^. MPV, which is the most frequently used platelet size measurement, is also an index of platelet activation^(34)^. Platelets release thrombin, which plays a role in inflammation^(34)^ and angiogenesis^(35)^, and a high platelet volume allows greater coagulability and fibrinolysis^(36)^. However, there is a limited number of studies about the relationship between MPV and the severity of ICP, and the relationship between MPV and perinatal outcomes, even though an MPV increase can be seen in patients with ICP^(31,32)^. Kebapcilar et al.^(31)^ investigated the relationship between coagulation parameters and low 5-minute Apgar scores in both patients with ICP and normal pregnancies. In addition, Oztas et al.^(32)^ reported higher MPVs in patients with ICP when compared with a control group, with an increased preterm delivery likelihood just after exceeding an MPV of 11.2 fL. In our study, MPV was significantly increased in women with ICP cases compared with healthy controls. Moreover, apart from the PLR and WBC, the MPV levels increased in the severe group.

### Study Limitations

The main limitation of this study was its retrospective design, but despite this methodologic limitation, this research investigated all of the available hematologic inflammatory parameters. It confirmed prior data that inflammatory markers are significantly increased in patients with ICP. Despite the significant changes in the inflammatory markers in patients with ICP, only MPV was significantly increased with the severity of the disease. These results suggest that MPV may be a valuable marker in patients with severe ICP, but large scale studies are needed to confirm this result.

## CONCLUSION

Based on the results of this study, the inflammatory markers were significantly increased in patients with ICP. PLR, WBC, and MPV were all significantly increased, whereas the RDW was significantly decreased in ICP. MPV was related to the severity of disease and might be a valuable marker for ICP disease severity in the future.

## Figures and Tables

**Table 1:**
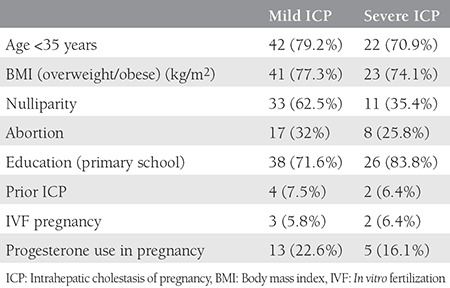
The basal characteristics of mild (n=53) and severe (n=31) intrahepatic cholestasis of pregnancy

**Table 2:**
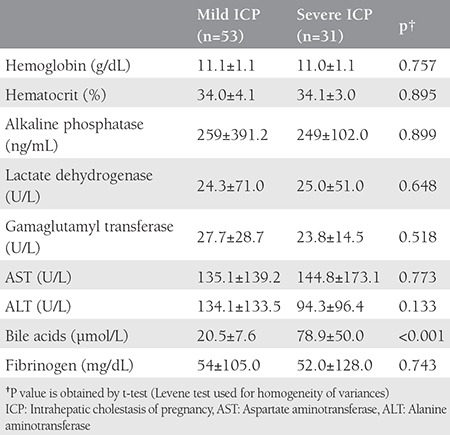
The comparison of the laboratory findings between patients with mild and severe intrahepatic cholestasis of pregnancy

**Table 3:**
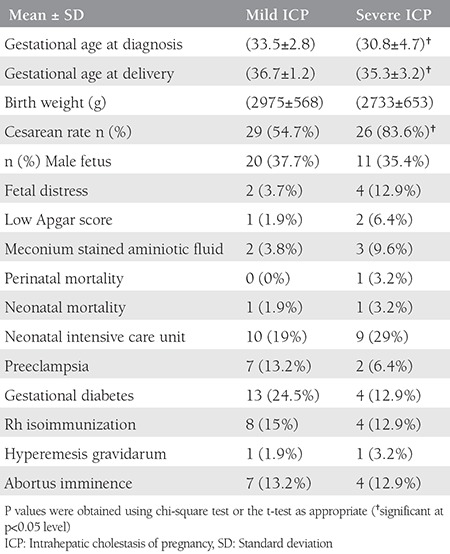
The comparison of obstetric outcomes in mild (n=53) and severe (n=31) intrahepatic cholestasis of pregnancy

**Table 4:**
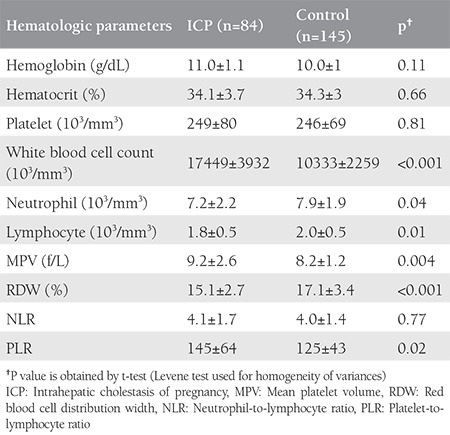
The comparison of hematologic indices between control and intrahepatic cholestasis of pregnancy group

**Table 5:**
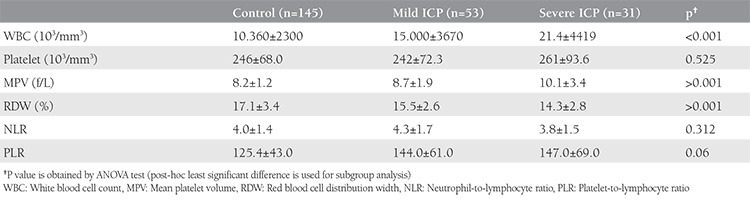
The comparison of inflammatory markers
